# Diversity and Resistance Profiles of ESBL-Producing Gram-Negative Bacteria from Dairy Farms in Southern Türkiye

**DOI:** 10.3390/antibiotics13121134

**Published:** 2024-11-25

**Authors:** Cemil Kürekci, Murat Yüksel, Büşra Gülay Celil Ozaslan, Sait Tan, Claudia Jäckel, Mirjam Grobbel, Jens Andre Hammerl

**Affiliations:** 1 Department of Food Hygiene and Technology, Faculty of Veterinary Medicine, Hatay Mustafa Kemal University, Antakya 31060, Türkiye; 2 Department of Obstetrics and Gynecology, Faculty of Veterinary Medicine, Hatay Mustafa Kemal University, Antakya 31060, Türkiye; murat.yuksel@mku.edu.tr; 3 Department of Microbiology, Faculty of Veterinary Medicine, Hatay Mustafa Kemal University, Antakya 31060, Türkiye; busragulay.celil@gmail.com; 4 Yavuzeli District Directorate of Agriculture and Forestry, Ministry of Agriculture and Forestry, Gaziantep 27060, Türkiye; saittan0221@gmail.com; 5 Department of Biological Safety, German Federal Institute for Risk Assessment, Max-Dohrn Str. 8-10, D-10589 Berlin, Germany; claudia.jaeckel@bfr.bund.de (C.J.); mirjam.grobbel@bfr.bund.de (M.G.); jens-andre.hammerl@bfr.bund.de (J.A.H.)

**Keywords:** antimicrobial resistance, One Health, dairy farms, plasmids, beta-lactams, transmission

## Abstract

**Background/Objectives:** The increasing occurrence of extended-spectrum β-lactamase (ESBL)–producing *Enterobacterales*, most commonly *Escherichia coli*, has become a serious problem. The aim of this study was to determine the presence of ESBL-producing Gram-negative bacteria in dairy cattle, goat and sheep farms located in southern Türkiye. **Methods:** Samples (409 quarter milk samples and 110 fresh faecal samples from cattle, 75 bulk tank milk samples and 225 rectal swab samples from goats and sheep) were subjected to selective isolation on MacConkey agar with ceftazidime (2 µg/mL). Isolates were identified by MALDI-ToF MS. The antimicrobial susceptibility profile of the isolates was determined by the broth microdilution method. To obtain a deeper insight into the genetic diversity of isolates substantially contributing to an efficient spread of their ESBL-determinants (23-MO00001: an *E. coli* from mastitis and 23-MO00002 *Citrobacter freundii*), the transmission potential and the genetic background of the plasmid carrying the *bla*_CTX-M_ determinant was studied with whole genome analysis using Illumina sequencing. **Results:** Of the samples tested, 47 from the bovine faecal samples, 1 from the subclinical mastitis milk sample, 9 from the goat/sheep rectal swab samples and 5 from the goat/sheep bulk tank milk samples had ceftazidime-resistant Gram-negative strains with the ESBL phenotype. Of the 33 ESBL-producing *E. coli* isolates, 66.6% were resistant to tetracycline, 57.6% to sulfamethoxazole, 48.9% to nalidixic acid, 42.4% to ciprofloxacin and 33.3% to trimethoprim. Pulsed field gel electrophoresis (PFGE) results showed that the majority of *E. coli* isolates (16/33) and all *Enterobacter* spp. isolates (n = 5) were not clonally related (80% similarity cut value). The sequenced strains were observed to efficiently transfer their ceftazidime resistance to the recipient strain *E. coli* J53 at 37 °C (transfer rates: 10^1^–10^2^ transconjugants per donor cell). S1-PFGE showed that the transconjugants J53(p23MO01-T1) and J53(p23MO02-T1) had acquired plasmids of about 82 kb and 55 kb plasmids, respectively. According to WGS results, the *E. coli* isolate was assigned to ST162, while the *C. freundii* isolate was assigned to ST95. **Conclusions:** This study demonstrates that dairy animals are reservoirs of ESBL-producing bacteria.

## 1. Introduction

Since antibiotics were approved and registered for use in food-producing animals commercially in the 1950s, they have been widely utilized as feed additives for growth promotion and disease prevention, as well as for the treatment of illnesses in livestock and aquaculture farming practices [1]. However, it is evident that improper and intensive usage of antibiotics in agriculture has inevitably raised significant environmental and human health concerns due to the development of resistance in pathogens under the selection pressure, which is also responsible for considerable medical costs [2,3]. It is estimated that 1.27 million deaths per year can be attributed to multidrug-resistant (MDR) bacterial infections [4]. Notwithstanding the cessation of the use of antibiotics as a growth-promoting agent in livestock in numerous countries, the projected global consumption of antibiotics in food-producing animals is expected to increase from 93,309 tons in 2017 to 104,079 tons by 2030 [5].

Beta (β)-lactam antibiotics have been widely utilized in dairy veterinary medicine for the management of various diseases, particular environmental mastitis, which is responsible for extensive economic losses on dairy farms in numerous countries [6,7]. In recent years, the emergence and spread of extended spectrum β-lactamase (ESBL)–producing *Escherichia coli* (*E. coli*) in food-producing animals have become a significant concern [8]. ESBLs are a group of hydrolytic enzymes that can act by hydrolysis of third-generation cephalosporins, penicillins and monobactams [9]. To date, a vast number of ESBLs have been discovered, with CTX-M enzymes being the most frequently detected types. As of July 2024, 270 variants of CTX-M have been reported [10]. It is noteworthy that a significant association has been identified between human clinical samples and the isolation of CTX-M-15-producing *E. coli*, particularly the ST131 clonal group [11]. Food-producing animals, especially poultry, are likely reservoirs for CTX-M-producing *E. coli* worldwide [12], necessitating the evaluation of potential risks to public health.

Several studies have demonstrated the presence of ESBL-producing *E. coli* in dairy herds, unpasteurized milk from cows, goats and sheep, milk products and even the environment of the animals (reviewed by [13]). In Türkiye, Aslantaş et al. (2017) observed an 8.3% carriage rate of ESBL-producing *E. coli* in healthy cattle [14]. As indicated by our previous investigations, ESBL-producing *E. coli* was present in 22.6% of the cow’s bulk tank milk samples [15]. With respect to ESBL enzymes, the CTX-M-15 type was rather common among isolates obtained from the milk samples [15]. However, further studies need to be carried out at the molecular level to gain deeper insights into the resistance mechanisms and epidemiology through whole genome sequencing (WGS). This study aimed to contribute to the documentation and understanding of the distribution of ESBL-producing *Enterobacterales* isolates in dairy farms as well as to provide valuable insights into the genetic characteristics of ESBL-producing *E. coli* and *Citrobacter freundii* (*C. freundii*) isolates through WGS in Türkiye.

## 2. Results

### 2.1. Detection of ESBL-Producing Enterobacterales on Dairy Farms

Of the 263 lactating cattle tested, 74.5% of the animals exhibited abnormal milk with the California Mastitis Test (CMT) scores of 1+, 2+, or 3+ in at least one-quarter of the individual udder. Furthermore, of the 409 milk samples obtained from the udder quarters of these animals, only one (0.25%) was culture-positive for ceftazidime-resistant Gram-negative bacteria. The isolate was reliably identified as *E. coli* using MALDI-ToF MS yielding to measurement scores of >2.3, indicating a very good prediction of the detected bacterium at species level.

Of the 110 faecal samples analysed, 47 were found to be positive for ceftazidime-resistant Gram-negative bacteria. The most prevalent species identified was *E. coli* (n = 45), followed by *C. freundii* (n = 1) and *Pseudomonas composti* (n = 1) across the four cattle farms. The lowest prevalence was observed on ‘Farm 2’ (1/18, 5.6%), while ‘Farm 4’ exhibited the highest prevalence (20/28, 71.4%). A comparison of the prevalence of *E. coli* in different age groups revealed a significantly higher incidence in calves (29/43, 67.4%) compared to adult cows and bulls (13/46, 28.3% and 5/21, 23.8%, respectively).

A total of 75 bulk tank milk samples from ovine and caprine farms were examined. Of these, five samples (5.3%) yielded ceftazidime-resistant Gram-negative bacteria. Three isolates were identified as *Acinetobacter* spp. (*Acinetobacter lactucae*, *A. pittii* and *A. baumannii*), while one was *E. coli* and one was *Enterobacter hormaechei*. Of the 225 rectal swab samples, 4% (n = 9) yielded ceftazidime-resistant Gram-negative bacteria, among which *E. coli* was identified in four samples: *Enterobacter hormaechei* (n = 1), *Pseudomonas aeruginosa* (n = 1), *Enterobacter kobei*/*roggenkampii* (n = 1) and *Enterobacter asburiae* (n = 1).

### 2.2. Antimicrobial Resistances and Genetic Diversity of Isolates at Farm Level

The general resistance profiles of the isolates were determined by antimicrobial susceptibility testing (AST). A set of 33 nonduplicate *E. coli* isolates were included for the further analysis and the remaining 18 were excluded because of their origin (from the same cattle farm and the same animal age group) and the similar antimicrobial resistance profile. The analysis of MIC results revealed that 33 *E. coli* isolates from all samples were found to be resistant to cefotaxime >4 µg/mL, ampicillin >32 µg/mL, ceftazidime (2 to >8 µg/mL). Of the 33 ESBL-producing *E. coli* isolates, 66.6% showed resistance to tetracycline, 57.6% to sulfamethoxazole, 48.9% to nalidixic acid, 42.4% to ciprofloxacin, 33.3% to trimethoprim, 18.2% to amikacin and 12.1% to gentamicin. However, none of them were resistant to tigecycline and meropenem. The MIC results of the present study for *E. coli* and non-*E. coli* isolates are given in Supplementary Table S1.

**Figure 1:**
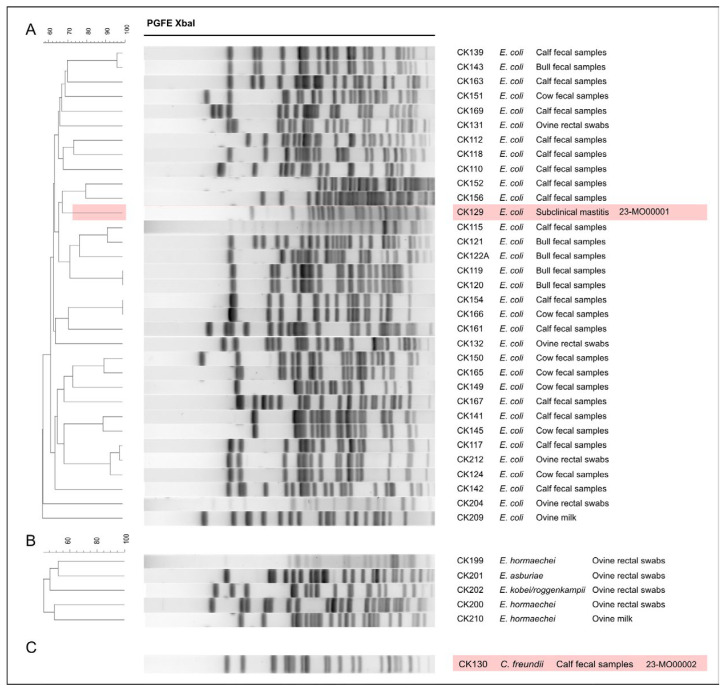
XbaI-macrorestriction profiles of isolates analysed in this study. Comparison of restriction profiles of (**A**) *E. coli*, (**B**) *Enterobacter* spp. and (**C**) the *C. freundii* isolate. The *E. coli* strain 23-MO00001 and *C. freundii* strain 23-MO00002 further subjected to WGS are indicated. The percentage of the phylogenetic relationship among the isolates is given on the left-hand side of the figure.

Clonal relation of all ESBL-producing *E. coli* and *Enterobacter* spp. isolates were analysed using the PFGE band profile (Figure 1) and it was found that 16 *E. coli* isolates displayed singletons, whereas 17 isolates were found to be related with a maximum three isolates clustered together (80% similarity cut-off value). Additionally, all *E. coli* isolates (except one; CK212) obtained from the goats/sheep samples were observed to be separated from those obtained in cattle farms. In addition, five *Enterobacter* spp. isolates were also found to be distinct from each other.

### 2.3. ESBL Determinants Are Encoded on Transmissible Plasmids

The potential of the isolates of this study for the spread of their ESBL phenotype was determined by in vitro filter mating studies. Out of the experiments, two strains were especially observed to efficiently transfer their ceftazidime resistance to the recipient strain *E. coli* J53 (transfer rates: *E. coli* donor 23-MO00001: 3.3 × 10^1^ and *C. freundii* donor 23-MO00002: 2.1 × 10^2^ transconjugants per donor cell). In this study, the main aim was to characterize ESBL-producing bacteria from subclinical mastitis cases, and ESBL-producing *C. freundii* strains have been increasingly reported in human clinical cases in many countries, hence it is of great importance in a public health point of view. Altogether, these isolates were therefore further subjected to in-depth analysis to evaluate their impact for the spread of their ESBL determinants. The minimum inhibitory concentration (MIC) results for the donor and transconjugant strains are presented in Table 1.

**Figure 2:**
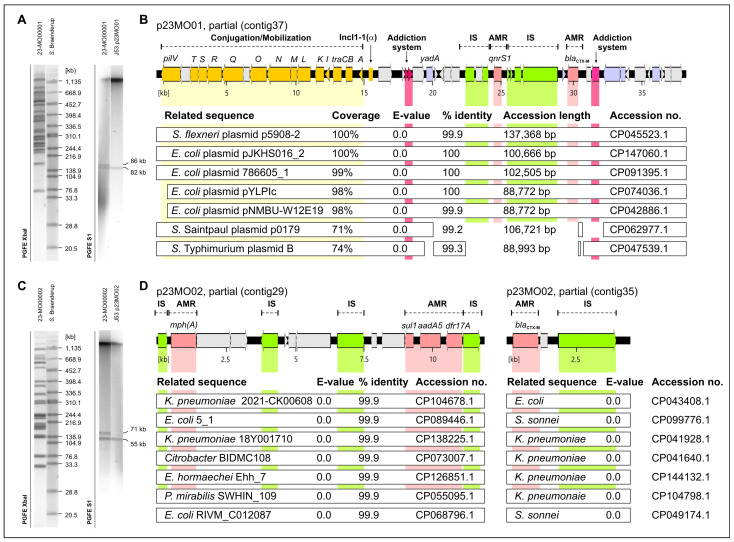
Determination of plasmids encoding ESBL resistance in *E. coli* strain 23-MO00001 and *C. freundii* strain 23-MO00002. (**A**,**C**) Results of XbaI-macrorestriction analysis (left) and S1 nuclease plasmid profiling (right) of the *E. coli* strain 23-MO00001 and the *C. freundii* strain 23-MO00002. As a reference, the XbaI-macrorestriction profile of the *Salmonella* Braenderup strain H9812 was used. (**B**,**D**) Determination of the antimicrobial resistance determinant encoding regions of plasmid p23MO01 and p23MO02. Genmaps indicate the organization of the respective regions of both plasmids. Genes are coloured according to their functional prediction: yellow, transfer genes; deep red, addiction system components; green, mobile genes (IS-elements, transposases), light red, antimicrobial resistance genes; violet, genes of other functions and grey, hypothetical genes. Below the genmaps, comparison of the sequence with publicly available plasmid genomes are given. White bars represent the homologous region covered by previously published plasmid sequences. Parameters such as coverage, e-value and % identity indicate the degree of the relationship to the individual plasmid genomes.

The transconjugants (J53(p23MO01-T1) and J53(p23MO02-T1)) exhibited resistance to cefotaxime (>4 µg/mL), ampicillin (>32 µg/mL) and ceftazidime (≥8 µg/mL). The recipient of the plasmid p23MO01 (donor *E. coli* 23-MO00001) also exhibited resistance to ciprofloxacin (0.5 µg/mL), whereas the recipient of p23MO02 (donor *C. freundii* 23-MO00002) demonstrated resistance to azithromycin (>64 µg/mL), trimethoprim (>16 µg/mL) and sulfamethoxazole (>512 µg/mL). S1-PFGE demonstrated that the transconjugants J53(p23MO01-T1) and J53(p23MO02-T1) had acquired plasmids of approximately 82 kb and 55 kb, respectively (Figure 2A,C).

The WGS sequencing results identified the *bla*_CTX-M-15_ gene, located on contigs mapped to plasmids, in both isolates belonging to the ESBL genotypes. Furthermore, *E. coli* isolate 23-MO00001 exhibited the presence of multiple antimicrobial resistance determinants, including *acrF*, *blaEC*, *emrD*, *mdtM* and *qnrS1* (Figure 2B). In contrast, *C. freundii* isolate 23-MO00002 demonstrated the carriage of the *bla*_CMY_, *mph(A)*, *sul1*, *aadA5* and *dfrA17* genes, which were located on contigs that were either mapped to chromosomes or plasmids (Figure 2C). The *E. coli* isolate from the milk sample was identified as ST162, while the *C. freundii* isolate from the calf faecal sample was classified as ST95.

It is noteworthy that the WGS results also revealed the presence of the IncFIB(pB171), IncFIA(HI1), IncFIC(FII) and IncI1-I(Alpha) replicon types in the *E. coli* genome, whereas the *C. freundii* genome exhibited only the IncFII plasmid replicon type.

A contig carrying *bla*_CTX-M-15_ assemblies of *E. coli* isolate had hits with query coverage of 100% and percent identity of 100% against *E. coli* plasmid pJKHSO16_2 (accession number CP147060.1) (Figure 2B). *C. freundii* had contig carrying *bla*_CTX-M-15_ that mapped to several plasmids of different bacterial species with 99.9% identity (Figure 2D). Apart from *bla*_CTX-M-15_, the plasmid p23MO01 contains also *qnrS1* responsible for quinolone resistance, whereas the plasmid p23MO02 contains *sul1*, *aadA5* and *dfrA17* genes responsible for sulfamethoxazole, aminoglycoside and trimethoprim resistance, respectively.

## 3. Discussion

A comprehensive analysis of scientific data revealed significant geographic variations in the prevalence of subclinical mastitis on dairy cattle farms worldwide [16]. For instance, a meta-analysis revealed that the prevalence of subclinical mastitis was 48.2% in African countries [17], while another meta-analysis indicated a prevalence of 42% globally [18]. The current study revealed that 74.5% of the animals exhibited abnormal milk with CMT scores, indicating a considerable prevalence of subclinical mastitis in the region.

The presence of ESBL-producing Gram-negative bacteria in the samples of clinical and subclinical mastitis, as well as in the bulk tank milk samples, was monitored in various studies (reviewed by [13]). In this study, only one-quarter of the milk samples tested positive for the presence of ESBL-producing *E. coli*. The colonization rate varies significantly based on a number of factors, particularly the type of mastitis. However, a low prevalence of ESBL-producing Gram-negative bacteria, including *E. coli*, has been reported in subclinical mastitis cases in many parts of the world (reviewed by [13]). It is noteworthy that only 5.3% of ovine and caprine bulk tank milk samples were found to be contaminated with ESBL-producing Gram-negative bacteria. In a previous study conducted in the same region, a considerably higher contamination rate (22.6%) of ESBL-producing *E. coli* was observed in bulk tank milk samples from cattle [15]. Similarly, Obaidat and Gharaibeh (2022) [19] also identified the presence of *bla*_CTX-M-15_-bearing *E. coli* in 33.5% of sheep and goat milk samples in Jordan. However, a considerably lower contamination rate (9.5%) was documented in bulk tank milk samples in Germany [20]. As previously observed [20], non-*E. coli* ESBL producers were more prevalent in bulk tank milk samples. To the best of our knowledge, we documented the presence of ESBL-producing *Acinetobacter* spp. in small ruminant bulk tank milk samples for the first time in Türkiye.

A substantial body of research has demonstrated that the faecal carriage of ESBL-producing bacteria in cattle farms exhibits considerable variability. The observed variations in the occurrence rate have been linked to a multitude of factors, including but not limited to age, breeding habits and production types [21]. The overall prevalence of faecal carriage in cattle farms was 42.7%, with substantial inter-farm variability (5.5–71.42%). A study conducted in Hatay province investigated the prevalence of ESBL/AmpC-producing *E. coli* in healthy cattle and reported an 8.3% prevalence rate without enrichment [14]. In alignment with prior findings [22], the prevalence of faecal carriage of ESBL-producing *E. coli* was high (67.4%) among calves. The beta-lactam group of antibiotics has long been employed to control mastitis in dairy farms, resulting in a considerable quantity of mastitis milk samples with antibiotic residue. Such discrepancies may be attributed to disparate methods of waste milk management employed on the various farms [23]. Furthermore, the transformation of the calf’s gastrointestinal tract and the subsequent alteration of its microbial flora may also contribute to the contamination process [22].

Conversely, 4% of faecal samples obtained from ovine/caprine species tested positive for ESBL-producing isolates. Among the bacteria detected were those of the *E. coli* species, together with *Enterobacter hormaechei*, *Pseudomonas aeruginosa*, *Enterobacter kobei*/*roggenkampii* and *Enterobacter asburiae*. In a recent study, Ramaztla et al. (2024) examined a single stool sample from each of the goats and sheep on four farms in South Africa [24]. They found that 28.3% of the samples were contaminated with ESBL-producing *E. coli*.

Furthermore, this study illustrated that ESBL-producing *E. coli* isolates exhibited resistance to a range of antibiotic classes, including tetracycline, sulfamethoxazole and ciprofloxacin. The high level of resistance to tetracycline may be attributed to the long-term and widespread use of this antibiotic on dairy farms in Türkiye. Additionally, a high level of resistance to tetracycline and ciprofloxacin was observed in ESBL-producing *E. coli* isolates in the United States [25]. The observed resistance to ciprofloxacin and sulfamethoxazole antibiotics has been linked to the co-location of corresponding resistance traits on the same mobilizable plasmid [26].

There was considerable genetic variation within ESBL-producing *E. coli* isolates using PFGE, which is consistent with the findings of Salaheen et al. (2019) [25]. In that study, isolates of *E. coli* from mastitis exhibited 27 unique clusters of digest patterns when an 80% similarity cut-off was used for cluster analysis.

In this study, two ESBL-producing isolates underwent WGS analysis. The production of ESBLs was found to be encoded by the *bla*_CTX-M-15_ gene, which was confirmed to be located on mobilizable plasmids in both species. In previous studies, the occurrence of ESBL production in *E. coli* has been commonly linked to the presence of ESBL enzymes, predominantly CTX-M-15 type, in food-producing animals [14,15]. The *bla*_CTX-M-15_ gene is typically located on a variety of plasmid incompatibility groups, including IncA, IncC, IncF, IncH, IncN, IncY, IncK, IncX, IncI, IncL and IncM [26].

In the current study, genomic analysis revealed that ESBL-producing *E. coli* from the subclinical mastitis case assigned to ST162. Fuentes-Castiollo et al. (2020) [27] showed the worldwide distribution of *E. coli* ST162 in different sources using the EnteroBase data, when analysing ESBL-producing *E. coli* ST162 from birds. Of note, *C. freundii* isolate assigned to ST95. In a recent study, analysis of clonal lineages of carbapenemase-producing isolates of the *Citrobacter* species revealed that imipenemase (IMP)-producing isolates were found to be mainly related to ST95 worldwide [28].

Even though collecting the samples in a restricted area in Türkiye is the primary limitation of this study to generalize these results, the presence of ESBL-producing Gram-negative bacteria, particularly *E. coli* strains, in dairy farms indicates a potential public health problem. Therefore, the results emphasize the importance of monitoring the occurrence of ESBL-producing bacteria in food-producing animals and the environment under the One Health perspective.

## 4. Materials and Methods

### 4.1. Sampling

This study was conducted on 50 dairy herds (46 ovine/caprine and 4 bovine (Farm A to D)) situated in southern Türkiye (Gaziantep and Hatay provinces). The mean herd size on bovine farms in Hatay province was n > 100. The samples were obtained from healthy lactating animals during routine udder health examinations. In these bovine herds, quarter milk samples from 263 lactating cattle in all stages of lactation were collected and tested with CMT on the farms. The milk samples were collected during the morning milking period, with the teats cleaned with 70% ethanol and the initial foremilk streams discarded. The CMT scores were interpreted by the authors as follows: negative (no precipitation), 1+ (mild; a distinct precipitate but no gel formation), 2+ (moderate; distinct gelatinization) and 3+ (heavy; distinct and thick gelatinization), as described by Barnum and Newbould (1961) [29]. All quarter milk samples with CMT scores of 1+, 2+ and 3+ (n = 409) were transferred to the laboratory on ice for microbiological examination. Furthermore, a total of 110 fresh cattle faecal samples were collected from the ground on the farms. The samples included 46 dairy cows and young stock, 43 calves younger than two months and 21 bulls.

In caprine and ovine herds, a total of 6300 sheep and 4700 goats were observed. A total of 225 rectal swab samples were collected from sheep (n = 88) and goats (n = 137). Furthermore, a total of 75 bulk milk samples were collected for microbiological examination, comprising 18 sheep, 37 goats and 20 mixed samples.

### 4.2. Isolation of Ceftazidime Resistant Gram-Negative Bacteria and Antimicrobial Resistance Determination

For the purpose of selective isolation, each swab sample was enriched in Enterobacteriaceae Enrichment (EE) broth for an overnight period at 37 °C. Additionally, the milk samples (1 mL) were enriched in EE broth for an overnight period at 37 °C. Subsequently, 10 µL of the enriched samples were streaked out on MacConkey agar, which had been supplemented with ceftazidime at a concentration of 2 µg/mL. The plates were incubated at 37 °C for 24 h. Following incubation, lactose-fermenting red-coloured colonies and non-lactose-fermenting colourless colonies were selected and purified on blood agar at 37 °C for 24 h. MALDI-ToF MS (Bruker, Biotyper, Bremen, Germany) was employed to determine the species of the selected isolates. According to the recommendation of the manufacturers, the direct transfer method was used with HCCA matrix (Bruker). The results obtained were interpreted according to the scores as ‘>2.300, highly probable species identification; 2.000–2.299, secure genus identification, probable species identification; 1.700–1.999, probable genus identification; and <1.700, not reliable identification’. The strains were stored in Microbank beads (Pro Lab Diagnostics Inc., Richmond Hill, ON, Canda) at a temperature of −80 °C.

AST was conducted by broth microdilution according to the CLSI guideline [30]. The minimum inhibitory concentrations (MICs) of ceftazidime-resistant Gram-negative isolates and transconjugants were determined by the use of Sensititre™ plates (EUVSEC3; Fisher Scientific, Loughborough, UK). The antimicrobials and concentrations tested are as follows: ampicillin (1–32 μg/mL), meropenem (0.03–16 μg/mL), ciprofloxacin (0.015–8 μg/mL), azithromycin (2–64 μg/mL), amikacin (4–128 μg/mL), gentamicin (0.5–16 μg/mL), tigecycline (0.25–8 µg/mL), chloramphenicol (8–64 μg/mL), colistin (1–16 μg/mL), nalidixic acid (4–64 μg/mL), tetracycline (2–32 μg/mL), trimethoprim (0.25–16 μg/mL) and sulfamethoxazole (8–512 μg/mL). The *E. coli* strain ATCC 25922 was employed as control strains for quality control of AST. The MIC values were interpreted in accordance with the Clinical and Laboratory Standards Institute (CLSI) criteria [30]. The production of ESBLs was confirmed through the application of double disc synergy and disc combination tests, in accordance with the CLSI guidelines [30].

### 4.3. Macrorestriction Profiling and Plasmid Prediction by PFGE

Genomic DNA was prepared using the standard PulsNet PFGE protocol for *E. coli* and other Enterobacterales bacteria (https://pulsenetinternational.org/protocols/pfge/, accessed on 1 January 2023). Agarose plugs were prepared using colony material from individual strains grown on LB agar (16–20 h at 37 °C) in CSB (cell suspension buffer; 100 mM Tris:100 mM EDTA, pH 8.0) adjusted to an optical density of 1.2–1.4 (OD 600 nm). Equal volumes (300 µL) of bacterial suspensions in CSB and 1% Sea-Kem Gold agarose were mixed and applied to PFGE plug moulds (Biorad, Feldkirchen, Germany). After solidification, the plugs were removed from the moulds and treated with proteinase K (20 mg/mL) in cell lysis buffer (50 mM Tris:50 mM EDTA, pH 8.0 + 1% sarcosyl) for 2 h at 56 °C, followed by two washes with water and TE buffer, respectively. One-quarter of the plugs was sliced and digested with XbaI (25 enzyme units/plug) restriction enzyme (29 *E. coli*) or S1 nuclease (4 enzyme units/plug) (an *E. coli* from a bovine milk sample (23-MO00001) and a *C. freundii* strain from a bovine faecal sample including representative colonies of their transconjugants from in vitro filter mating studies). PFGE analysis was performed on a CHEFDRIII SYS220/240 system (Bio-Rad Laboratories GmbH, Munich, Germany). *Salmonella* serotype Braenderup strain H9812 was used as a control according to the PulsNet protocol (https://pulsenetinternational.org/protocols/pfge/, accessed on 1 January 2023). PGFE analysis was performed using Bionumerics (v7.6.3; Applied Maths; Sint-Martens-Latem, Belgium). The resulting XbaI and S1 profiles were analysed, recording the presence/absence of fragments >20 kb. Genetic similarity between profiles was determined using the unweighted pair method (UPGMA) with the Dice coefficient (settings: optimisation: 0.5%, band matching tolerance: 0.5%; other settings were used with default parameters).

### 4.4. Plasmid Transmission Evaluation

Plasmid transmission analysis was conducted by in vitro filter mating examination using *E. coli* 23-MO00001 from subclinical mastitis and *C. freundii* 23-MO00002 as donors, and the *E. coli* strain J53 (SAZ^R^: sodium azid-resistant) as the recipient. For mating of the bacteria, overnight cultures of the donors and recipient were mixed at a ratio of 1:2 and subjected to centrifugation at 4000× *g* for 4 min. The bacterial pellet was resuspended in 100 µL of lysogeny broth (LB) and applied onto a 0.22 µm pore-size filter on a LB agar plate. After an incubation at 37 °C for 4 h, the filters were removed and resuspended in 4 mL LB, out of which 100 µL were applied onto an LB agar plate supplemented with sodium acid (SAZ: 200 µg/L) and cefotaxime (FOT: 16 µg/L) and incubated for 16–20 h at 37 °C. Appearing colonies were picked onto double selective LB agar (SAZ/FOT) plates and subjected to MALDI-TOF MS and AST for final evaluation.

### 4.5. Whole-Genome Sequencing and Bioinformatics Analysis

Genomic DNA was extracted using the PureLink Genomic DNA extraction Kit (Invitrogen, Darmstadt, Germany) according to the recommendation of the manufacturers for Gram-negative bacteria. A DNA library prepared with the Nextera DNA Flex kit (Illumina, San Diego, CA, USA) was sequenced in 2 × 151 cycles on an Illumina NextSeq 500 sequencer using the NextSeq 500/550 midoutput kit v2.5. Raw reads were subjected to the Aquamis pipeline (https://gitlab.com/bfr_bioinformatics/AQUAMIS, accessed on 1 January 2024) for trimming, de novo assembly and quality evaluation [31]. If not stated otherwise, the software was used with default settings. In silico typing was conducted using the BfR in-house Bakcharak pipeline (v3.0.3: source code of the software is provided at https://gitlab.com/bfr_bioinformatics/bakcharak), which utilized the software abricate (v1.0.1), AMRfinder (v3.10.45) [32], the VFDB [33], plasmidfinder (v2.1) [34], platon (v1.6), mash (v2.3) and snakemake (v7.17.1). The PubMLST scheme was employed for the detection of MLST (v2.22.0). Further analysis of individual contigs (i.e., contigs carrying *bla*_CTX-M_) were subjected to analysis using DS Gene (v2.5, Accelrys Inc., San Diego, CA, USA). For the evaluation of the relationship of plasmidic contigs the BLASTn suite (Core Nucleotide Database (core_nt)) of the National Center for Biotechnology Information (v2.12.0, access: August 2024) was used.

Assemblies of the genomes of the *E. coli* strain 23-MO00001 (biosample: SAMN43244124) and the *C. freundii* strain 23-MO00002 (biosample: SAMN43244125) were submitted to GenBank (NCBI) as part of the bioproject PRJNA1149394.

## Figures and Tables

**Table 1:** Antimicrobial susceptibility testing of ESBL-producing Gram-negative isolates against different antimicrobials. MIC values resulting in non-wildtype phenotypes are indicated in grey.

Strain	AMP	AZI	CHL	CIP	COL	FOT	GEN	MER	NAL	SMX	TAZ	TET	TGC	TMP
*Ec* J53	≤4	≤2	≤8	0.03	≤1	≤0.25	≤0.5	≤0.03	≤4	≤8	≤0.25	≤2	≤0.25	≤0.25
*Ec* 23-MO00001	>32	4	≤8	0.5	≤1	>4	≤0.5	≤0.03	8	≤8	4	≤2	≤0.25	≤0.25
*Ec* (p23MO01T)	>32	≤2	≤8	0.5	≤1	>4	1	≤0.03	≤4	≤8	8	≤2	≤0.25	≤0.25
*Cf* 23-MO00002	>32	>64	≤8	0.03	≤1	>4	1	0.06	≤4	>512	4	≤2	0.5	>16
*Ec* (p23MO02T)	>32	>64	≤8	0.03	≤1	>4	≤0.5	≤0.03	≤4	>512	>8	≤2	≤0.25	>16

Abbreviation: *Ec*, *E. coli*; *Cf*, *C. freundii*; AMP, ampicillin; AZI, azithromycin; CHL, chloramphenicol; CIP, ciprofloxacin; COL, colistin; FOT, cefotaxime; GEN, gentamicin; MER, meropenem; NAL, nalidixic acid, SMX, sulfamethoxazole; TAZ, ceftazidime; TET, tetracycline; TGC, tigecycline; TMP, trimethoprim. MIC values are given in mg/L.

## Data Availability

All data are available in the manuscript including the Supplemental Materials. The genomes of the *E. coli* strain 23-MO00001 (biosample: SAMN43244124) and the *C. freundii* strain 23-MO00002 (biosample: SAMN43244125) were deposited in Genbank (NCBI) in the bioproject PRJNA1149394.

## Supplementary Materials

The following supporting information can be downloaded at: https://www.mdpi.com/article/10.3390/antibiotics13121134/s1, Table S1: Minimum inhibitory concentrations of ESBL-producing Gram-negative bacteria from dairy farms.
